# Fentanyl Exposure in Preterm Infants: Five-Year Neurodevelopmental and Socioemotional Assessment

**DOI:** 10.3389/fpain.2022.836705

**Published:** 2022-03-01

**Authors:** Kimberly P. Mills, Rachel E. Lean, Christopher D. Smyser, Terrie Inder, Cynthia Rogers, Christopher C. McPherson

**Affiliations:** ^1^Department of Pharmacy, St. Louis Children's Hospital, St. Louis, MO, United States; ^2^Department of Psychiatry, Washington University in St. Louis, St. Louis, MO, United States; ^3^Department of Pediatrics, Washington University in St. Louis, St. Louis, MO, United States; ^4^Department of Neurology, Washington University in St. Louis, St. Louis, MO, United States; ^5^Department of Radiology, Washington University in St. Louis, St. Louis, MO, United States; ^6^Pediatric Newborn Medicine, Brigham and Women's Hospital, Boston, MA, United States

**Keywords:** premature infant, neonatal intensive care, analgesic, opioid analgesic, neurodevelopmental outcomes, socioemotional outcomes

## Abstract

**Objective:**

To evaluate the association between cumulative fentanyl dose during neonatal intensive care and 5-year neurodevelopmental and socioemotional outcomes in very preterm infants.

**Materials and Methods:**

Patient demographics and clinical factors during the perinatal and neonatal course were collected in 84 patients born between 23- and 30-weeks gestational age (GA). Cumulative fentanyl dose during neonatal intensive care was calculated. Developmental testing at age 5 years included the Wechsler Preschool and Primary Scale of Intelligence Full-Scale Intelligence Quotient, Third Edition, Clinical Evaluation of Language Fundamentals-Preschool, Second Edition, Movement Assessment Battery for Children, Second Edition (MABC-2), and Shape School Assessment. Socioemotional outcomes were assessed via caregiver's responses on the Child Behavior Checklist/1.5-5 (CBCL/1.5-5.5) and Social Responsiveness Scale, Second Edition (SRS-2). Covariates were identified on bivariate analysis (*p* < 0.1). Linear regression models related outcome measures to the log of cumulative fentanyl dose adjusted for covariates.

**Results:**

Higher cumulative fentanyl dose was associated with lower composite motor scores on bivariate analysis (*p* < 0.01). Cumulative fentanyl dose did not correlate with composite intelligence quotient, language, or executive function. The Clinical Risk Index for Babies score, log of mechanical ventilation, inotrope, and anesthesia duration, and log of cumulative midazolam and hydrocortisone dose were also associated with MABC-2 scores (*p* < 0.1). Cumulative fentanyl dose was not associated with composite MABC-2 scores on multiple linear regression. Higher cumulative fentanyl dose was associated with decreased socioemotional problems based on caregiver's response on CBCL/1.5-5.5 t-scores driven by fewer symptoms of depression. The McMaster Family Assessment Device general functioning scale score, maternal age, GA, log of total parenteral nutrition days, patent ductus arteriosus requiring treatment, and log of inotrope hours were also associated with CBCL/1.5-5.5 t-scores (*p* < 0.1). Cumulative fentanyl dose (*p* = 0.039) and family dysfunction score (*p* = 0.002) remained significant after controlling for covariates on multiple linear regression.

**Conclusion:**

Cumulative fentanyl dose during neonatal intensive care did not correlate with 5-year motor, cognitive, or language outcomes after controlling for other variables. Fentanyl dose was associated with caregiver reported total socioemotional problems on the CBCL/1.5-5.5 on multivariate modeling. Additional long-term studies are needed to fully elucidate the safety of fentanyl in very preterm neonates.

## Introduction

Very preterm infants are uniquely susceptible to the sensation of pain due to immature descending pain modulatory pathways in comparison to more mature ascending pain perception pathways (1). Neonatal stressors, including acute pain with inadequate analgesia, have been associated with an increased risk of brain injury including intraventricular hemorrhage (IVH), periventricular leukomalacia (PVL), and neuronal apoptosis (2, 3). Further, untreated pain in very preterm infants has been associated with reduced parietal brain volume and functional connectivity in the temporal lobes and poorer neurobehavioral outcomes at term equivalent (4). Pain-related stress in very preterm infants has also been associated with alterations in white matter microstructure, reduced cerebellar volume, and poorer cognitive outcomes at school age follow-up (5, 6). Opioids are often utilized prior to painful procedures and during prolonged mechanical ventilation in very preterm infants (7, 8). However, there are significant safety concerns regarding the use of opioids and their negative impact on the immature nervous system with resultant adverse short- and long-term neurodevelopmental outcomes (9).

Morphine is a natural opioid; its administration has been associated with clinically significant hypotension in very preterm infants, which may contribute to an increased risk of cerebral ischemia resulting in IVH and PVL. Large randomized controlled trials have assessed the short- and long-term neurological outcomes of morphine administration in mechanically ventilated very preterm infants reporting subtle and mixed long-term outcomes (9–12). In other observational studies, generally lower doses of morphine have been reported to be relatively safe (12–14), while higher cumulative doses appear to be associated with reduced cerebellar volume at term equivalent age and impaired motor development, cognitive function, and increased caregiver reported social problems (15, 16).

Fentanyl is a synthetic opioid that is hypothesized to have a lesser propensity for hypotension in very preterm infants (17, 18). Fentanyl use in the neonatal intensive care unit (NICU) is much more commonly reported compared to morphine, despite much more limited literature evaluating its pharmacokinetics and short- and long-term outcomes (7, 8). Small randomized controlled trials have shown that mechanically ventilated very preterm infants receiving continuous fentanyl infusions had reduced stress response with no difference in the incidence of IVH or PVL (19–21). However, a retrospective study of our cohort associated cumulative fentanyl dose during NICU care with an increased risk of cerebellar hemorrhage and a dose-dependent reduction in cerebellar diameter on magnetic resonance imaging (MRI) (22).

Preclinical models utilizing neonatal mice have demonstrated that prolonged opioid exposure significantly reduces brain volume with long-term consequences including motor delays (23). Long-term neurodevelopmental outcomes evaluating the safety of fentanyl in premature human infants are sparse, reporting only at 9 months and 2 years of age (22, 24, 25). No associations with socioemotional outcomes have been reported in relation to fentanyl exposure in the very preterm infant. It has been shown in very preterm infants that motor delays are also associated with reduced attention and lower social competence as early as 2 years of age with persistence to school age (26). Therefore, we assessed neurodevelopmental and socioemotional outcomes at 5 years of age in an observational cohort exposed to open-label fentanyl to assist in defining the long-term impact of cumulative fentanyl dose in very preterm infants. We hypothesized, based on our earlier observation in this cohort of a negative impact on cerebellar hemorrhage and growth, that cumulative fentanyl dose would be associated with neurodevelopmental delays and socioemotional problems.

## Materials and Methods

This observational cohort study included very preterm infants born between 23- and 30-weeks gestational age (GA) that were enrolled at birth following admission to the NICU at St. Louis Children's Hospital between April 2007 and June 2010. Infants with known congenital anomalies, cardiorespiratory failure (>80% FiO2 for 6 h and/or ≥2 inotropic drugs), or severe IVH (Grade III and IV) in the first 24 h of life were excluded. Cumulative doses of all analgesia and sedatives from birth until term equivalent age or prior to NICU discharge were collected from the electronic medication administration records. Patient demographics (sex, GA, birth weight), perinatal course [antenatal steroids, 5-min APGAR score, Clinical Risk Index for Babies (CRIB) score at birth (27)], and neonatal course [duration of total parenteral nutrition (TPN), ventilation, and inotropes, and cumulative doses of hydrocortisone and dexamethasone] were also collected. At the 5-year follow-up, social risk [an index based upon maternal age at delivery (<18 years), no high school qualification, single parent, African American race/ethnicity as a proxy for systemic and individual experiences of racial discrimination, and public health insurance], income-to-needs ratio, and the General Functioning scale score of the McMaster Family Assessment Device (higher scores indicate unhealthy functioning) were also collected to assess the socioeconomic and family environment (28, 29). Approval for ethical study conduct was obtained by the Washington University in St. Louis Institutional Review Board.

### Follow-Up Assessment at Age Five Years

Participants underwent developmental assessments of cognition, language, motor skills, and executive function at 5 years of age conducted by highly experienced psychometricians at the Washington University Intellectual and Developmental Disabilities Research Center. Child socioemotional problems were assessed through questionnaires completed by the child's primary caregiver. Contact with families of all surviving children was attempted, regardless of participation in the 2-year follow-up wave of this longitudinal study. The Wechsler Preschool and Primary Scale of Intelligence Full-Scale Intelligence Quotient, Third Edition (WPPSI-III) was used to assess cognition (30). The Clinical Evaluation of Language Fundamentals-Preschool, Second Edition (CELF-P2) was used to assess core language (31). The Movement Assessment Battery for Children, Second Edition (MABC-2) was used to assess fine and gross motor function (32). These valid and reliable neurodevelopmental tests have been used to identify intellectual, language, and motor delays in childhood (33–35). The Shape School Task was used to evaluate executive function. Efficiency scores (correct-errors/time) for the set shifting, cognitive inhibition, and inhibition-shifting switch conditions were recorded (36). The Shape School correlates with other neuropsychological measures of attention/executive function (37) and has been used in samples of very preterm children (38, 39). The primary caregiver of the child completed the Child Behavior Checklist/1.5-5 (CBCL/1.5-5.5) and the Social Responsiveness Scale-2 (SRS-2) questionnaires to assess socioemotional outcomes and autistic traits, respectively (40, 41). Mood/affective problems, somatic complaints, and social withdrawal were assessed on the Internalizing scale of the CBCL/1.5-5 assessment. Inattention/hyperactivity as well as aggressive behavior were evaluated via the Externalizing scale of the CBCL/1.5-5 assessment. The SRS-2 Total t-score is comprised of two subscales, the Social Communication and Interaction t-score which assesses social-communication problems and the Restricted and Repetitive Behaviors (RBR) t-score which assesses autistic mannerisms. Both the CBCL and SRS demonstrate excellent test re-test reliability, internal consistency, and predictive validity for psychiatric disorders in youth (42, 43).

### Statistical Analysis

Statistical analyses were performed in SPSS 25 (SPSS, Inc., Chicago, Illinois). Variables with skewed distribution were log transformed for statistical analysis. Association between log of cumulative fentanyl dose during the NICU stay and neurodevelopmental and socioemotional outcomes were assessed using linear regression models. The composite scores from the WPPSI-III, CELF-P2, and MABC-2 were used to assess cognitive, motor, and language outcomes, respectively. The CBCL/1.5-5.5 and SRS-2 were assessed as both composite and subscale scores. The Shape School Task was assessed via subscale scores. Potential covariates [maternal age, antenatal steroids, GA, birthweight, sex, 5-min APGAR score, CRIB score, log ventilation days, log TPN days, patent ductus arteriosus (PDA) requiring treatment, necrotizing enterocolitis, retinopathy of prematurity, chronic lung disease, IVH on cranial ultrasound using the Volpe grading system (44), PVL on cranial ultrasound, cerebellar hemorrhage on MRI (45), cerebellar diameter on MRI, log anesthesia hours, log inotrope hours, log morphine dose, log midazolam dose, log dexamethasone dose, log hydrocortisone dose, social risk composite, income to needs ratio, and McMaster Family Assessment Device general functioning scale score] for outcomes were identified on bivariate analysis. A *p* < 0.1 was used to identify potential covariates on bivariate analysis to more comprehensively assess the complement of factors that may contribute to neurodevelopmental and socioemotional outcomes in very preterm infants. Multivariate linear regression models were utilized to relate outcome measures to the log of cumulative fentanyl dose, after adjusting for potential covariates with *p* < 0.05 utilized to identify significant associations. For multivariate models, collinearity diagnostics were reviewed and all variance inflation factor (VIF) and tolerance values were found to be within or close to the acceptable range (VIF <5).

## Results

### Clinical and Social Background Characteristics

**Figure 1:**
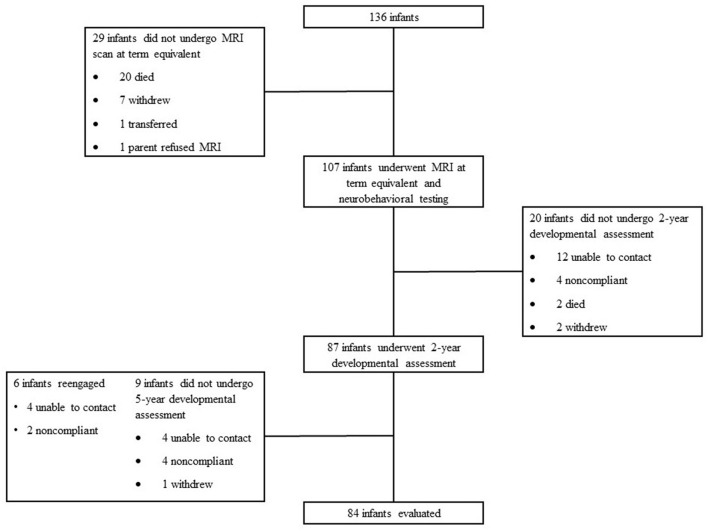
Subject selection and follow-up. One hundred and thirty-six infants were admitted to St. Louis Children's Hospital born between gestational ages ≥23 to ≤30 weeks. Twenty-nine infants did not undergo MRI scan at term equivalent; 20 died, 7 withdrew, 1 transferred, and 1 parent refused the MRI. One hundred and seven infants underwent MRI at term equivalent and completed neurobehavioral testing. Twenty infants did not undergo 2-year developmental assessment; 12 were not able to be contacted, 4 were non-compliant, 2 died, and 2 withdrew. Eighty-seven infants underwent 2-year development assessment. Nine infants did not undergo 5-year developmental assessment; 4 were not able to be contacted, 4 were non-compliant, and 1 withdrew. Six infants re-engaged at the 5-year assessment; 4 were previously not able to be contacted and 2 were previously non-compliant. Eighty-four infants were evaluated at 5 years for developmental assessment.

Eighty-four patients (80% of survivors in the overall cohort) were assessed at 5-year follow-up between August 2012 and March 2016 (Figure 1). Patients that were lost to follow-up were statistically more likely to be African American, had mothers with a younger age at delivery, and were more likely to be exposed to marijuana prenatally. They were also less likely to have private insurance and to have received antenatal steroids. Patient demographics, clinical outcomes, incidence of brain injury, medication exposure, and socioeconomic variables are presented in Table 1. Twenty-one patients (25%) did not receive any analgesia or sedative medications before term equivalent age or NICU discharge. The majority of patients that did receive analgesia or sedation were exposed to fentanyl; 62 patients (73.8%) were exposed prior to term equivalent age [median cumulative dose from birth to term equivalent age 1.9 mcg/kg, interquartile range (IQR) 0-307 mcg/kg, range 1-3,900 mcg/kg] (Table 1). Twelve infants (14.3%) received morphine; 4 patients (4.7%) received a total cumulative dose of >1 mg/kg; 11 infants (91.7%) were also exposed to fentanyl. Fifteen infants (17.9%) received midazolam; 5 patients (5.9%) received a total cumulative dose >1 mg/kg; 15 infants (100%) were also exposed to fentanyl.

**Table 1:** Characteristic of infants who underwent assessment at 5-years of age.

**Characteristic**	***n*** **= 84**
Gestational Age, weeks*^a^*	27 ± 2
Birth weight, g*^a^*	933 ± 256
Growth restriction for weight at birth	7 (8.3%)
Growth restriction for occipito frontal circumference at birth	12 (14.3%)
Male sex	42 (50%)
Antenatal steroids	67 (79.8%)
5-min APGAR score*^a^*	6.2 ± 2.0
Clinical Risk Index for Babies score*^a^*	4.1 ± 3.6
Ventilator days*^b^*	3 (1–18)
Total parenteral nutrition days*^b^*	17 (11–32)
Patent ductus arteriosus requiring treatment	39 (46.4%)
Necrotizing enterocolitis	6 (7.1%)
Retinopathy of prematurity	5 (6%)
Chronic lung disease	28 (33.3%)
Intraventricular hemorrhage on cranial ultrasound	33 (39.3%)
Grade I	6 (7.1)
Grade II	18 (21.4)
Grade III	2 (2.4%)
Grade IV	7 (8.3%)
Periventricular leukomalacia on cranial ultrasound	6 (7.1%)
Cerebellar hemorrhage on magnetic resonance imaging	17 (20.2%)
Grade I	8 (9.5%)
Grade II	4 (4.8%)
Grade III	3 (3.6%)
Grade IV	2 (2.4%)
Anesthesia exposure	18 (21.4%)
Inotrope exposure	19 (22.6%)
Fentanyl exposure	62 (73.8%)
Morphine exposure	12 (14.3%)
Midazolam exposure	15 (17.9%)
Dexamethasone exposure	8 (9.5%)
Hydrocortisone exposure	15 (17.9%)
Social risk composite*^b^*	1.5 (0-3)
Income to need ratio*^b^*	1.2 (0.8-2.4)
McMaster Family Assessment Device*^b^*	1.4 (1.1-1.8)

a *Mean ± standard deviation*;

b *Median (interquartile range)*.

### Neurodevelopmental Outcomes

Cognitive, language, and executive function testing were completed in all patients who returned for follow-up (Figure 1). Regarding the motor function assessment, 8 patients were unable to complete testing (7 patients) or were uncooperative (1 patient) (28). Cumulative fentanyl dose did not correlate with full-scale intelligence quotient, language, or executive function scores (Table 2). Higher cumulative fentanyl dose was associated with lower composite MABC-2 scores on bivariate analysis (Table 2). On bivariate analysis, CRIB score, GA, birth weight, log of ventilation days, log of inotrope and anesthesia hours, and log of cumulative midazolam and hydrocortisone dose were also independently associated with reduced composite MABC-2 scores (*p* < 0.1) (Supplementary Table 1). Birth weight and GA are components in the CRIB score, so they were excluded as covariates in the multiple regression model. However, similar associations were found on multivariate analysis when using birth weight and GA in place of CRIB score. Cumulative fentanyl dose was not associated with composite MABC-2 scores on multiple linear regression after adjusting for all covariate factors (Table 3).

**Table 2:** Composite neurodevelopmental outcomes in relation to log of cumulative fentanyl dose on bivariate linear regression.

**Variable**	**B**	**SE**	***R*** **2**	***P*** **-value**
MABC-2 Total t-score	−2.835	0.942	0.11	0.004
CBCL/1.5-5.5 Total Problems t-score	−2.629	1.365	0.046	0.058
Internalizing Problems t-score	−2.272	1.263	0.04	0.076
Externalizing Problems t-score	−2.623	1.265	0.053	0.041
WPPSI-III FSIQ score	−1.159	1.234	0.011	0.35
CELF-P2 Core Language score	−0.696	1.599	0.002	0.665
**Shape School Task**				
Shifting Efficiency	−0.015	0.024	0.079	0.532
Inhibition Efficiency	−0.067	0.039	0.044	0.092
Shifting/inhibition Efficiency	−0.012	0.03	0.003	0.684
SRS-2 Total t-score	−1.502	1.054	0.025	0.158
Social Communication and	−1.408	1.037	0.023	0.178
Interaction t-score				
Restricted and Repetitive Behaviors	−1.548	1.111	0.024	0.167
t-score				

*CBCL/1.5-5.5, Child Behavior Checklist/1.5-5; CELF-P2, Clinical Evaluation of Language Fundamentals-Preschool, Second Edition; MABC-2, Movement Assessment Battery for Children, Second Edition; SRS-2, Social Responsiveness Scale, Second Edition; WPPSI-III FSIQ, Wechsler Preschool and Primary Scale of Intelligence Full-Scale Intelligence Quotient, Third Edition*.

**Table 3:** Multivariate regression analysis of MABC-2 Total t-score.

**Variable**	**B**	**SE**	**Beta**	***R*** **1**	***P*** **-value**
Model				0.45	0.025
Log fentanyl dose	−0.508	2.291	−0.063		0.826
CRIB Score	−0.196	0.73	−0.061		0.79
Log ventilation days	−0.735	2.343	−0.109		0.756
Log anesthesia hours	0.079	0.913	0.016		0.932
Log inotrope hours	0.797	1.514	0.152		0.602
Log midazolam dose	−7.655	6.796	−0.226		0.268
Log hydrocortisone dose	−1.685	1.605	−0.277		0.302

*CRIB, Clinical Risk Index for Babies*.

### Socioemotional Outcomes

Socioemotional outcomes were obtained for 79 children (1 caregiver had limited English proficiency, 4 caregivers did not complete the questionnaire). Cumulative fentanyl dose did not correlate with autistic traits on the SRS-2 t-score, or either subscale including the Social Communication and Interaction t-score and RBR t-score. Cumulative fentanyl dose was associated with total socioemotional problems on caregiver reported CBCL/1.5-5 t-scores, driven primarily by higher levels of depressive symptoms in patients with low or no fentanyl exposure (Table 2; Supplementary Table 2; Supplementary Figure 1). Additionally, on bivariate analysis, the McMaster Family Assessment Device general functioning scale score, maternal age, GA, log of TPN days, PDA requiring treatment, and log of inotrope hours were also associated with total CBCL/1.5-5.5 scores (Supplementary Table 3). On multivariate analysis, the log of cumulative fentanyl dose and family dysfunction maintained significance after adjusting for all other covariate factors (Table 4).

**Table 4:** Multivariate regression analysis of CBCL/1.5-5.5 Total Problems t-score.

**Variable**	**B**	**SE**	**Beta**	***R*** **1**	***P*** **-value**
Model				0.713	0.001
Log fentanyl dose	−7.166	3.333	−0.572		0.039
McMaster Family Assessment Device	19.553	5.626	0.51		0.002
Maternal age	−0.468	0.342	−0.192		0.181
Gestational age	1.725	0.006	0.028		0.978
Log TPN days	5.15	0.012	0.053		0.958
PDA requiring treatment	−6.725	5.53	−0.198		0.233
Log inotrope hours	2.172	1.756	0.269		0.226

*PDA, patent ductus arteriosus; TPN, total parenteral nutrition*.

## Discussion

This is the first report of a cohort of very preterm children evaluating the association between cumulative neonatal fentanyl dose and neurodevelopmental and socioemotional outcomes at early school age. At 5 years of age in our cohort, cumulative fentanyl dose during NICU care was associated with motor delays on bivariate linear regression, but significance was not maintained on multivariate analysis. No other neurodevelopmental outcomes were associated with cumulative fentanyl dose. Cumulative fentanyl dose and family dysfunction were associated with total socioemotional problems via caregiver report on the CBCL/1.5-5.5, which remained significant after adjusting for covariate factors.

While inadequate analgesia in very preterm infants may have neurological consequences, opioids used to treat pain are not without risk. Opioid exposure during neonatal intensive care of very preterm infants has been associated with reduced brain growth and impaired long-term developmental outcomes. One previous study has reported a negative impact of morphine infusion during invasive mechanical ventilation on neurological outcomes at 8–9 years of age (14). Preclinical trials have shown that opioids can halt cellular proliferation and trigger apoptosis, with a particular affinity for Purkinje cells in the cerebellum (46, 47). This underlying pathophysiology is consistent with prior work from our group demonstrating an association between cumulative fentanyl dose and reduction in cerebellar diameter and increased risk of cerebellar hemorrhage in this patient cohort at term equivalent age (22). The cerebellum plays a key role in coordination, motor learning, and balance (48). Given the function of the cerebellum, an association of cumulative neonatal fentanyl dose and altered motor function at 5 years is plausible. Growing evidence has also recognized the role of the cerebellum in important non-motor functions. Impaired cerebellar development and cerebellar hemorrhagic injury in very preterm infants has been associated with altered social-behavioral, cognitive, executive function, and language development (49–51).

Long-term follow-up of motor performance is vital in cohorts of children born prematurely. Motor performance assessments have been validated in the first 2 years of life, but task performance can be affected by unintentional or abnormal motor signs commonly observed in very preterm infants in this age range. Furthermore, transient early dystonia may not preclude the development of typical long-term motor function in premature children (52). In this cohort, cumulative fentanyl exposure was not associated with motor outcomes at 2 years of age measured via the Bayley Scales of Infant and Toddler Development, Third Edition (22). Despite the longer time to follow-up, we had a similar number of participants at the 2-year timepoint for this cohort and for other studies where assessments were performed at 9 months and 2-years (22, 24, 25). In other cohorts of very preterm children, motor function was normal at 5 years in ~60% of infants born at a GA of ≤ 32 weeks (53, 54). In contrast, the rate of motor delay in our cohort was higher than this average, with less than half of patients having normal motor function at 5 years of age (28).

Cumulative fentanyl dose during the NICU stay was associated with motor delays in a dose-dependent manner on bivariate linear regression at 5 years of age in our cohort. However, it is important to understand the limitations of the observational nature of this study with the implicit challenges of the co-associations with cumulative fentanyl dose that may have neurological impact. This was apparent when the bivariate findings did not remain significant following adjustment for the many other covariates. On multiple linear regression accounting for the effects of identified covariates, the association between cumulative fentanyl dose and reduced composite MABC-2 scores was attenuated. In fact, no additional covariate factors that were associated with reduced composite MABC-2 scores in bivariate analyses maintained statistical significance in multiple linear regression analysis. These findings highlight the multifactorial and complex processes, that are often co-bedded, underlying neurodevelopmental outcomes in very preterm infants. Our findings are consistent with the current literature regarding neonatal fentanyl exposure and long-term neurodevelopmental outcomes (22, 24, 25). The null findings in our study support the hypothesis that fentanyl has the potential to impact long-term motor outcome, but exposure to even high doses may not independently modulate risk of impairment.

Cumulative fentanyl dose was independently associated with decreased socioemotional problems via caregiver report on the CBCL/1.5-5.5, and this finding persisted after adjusting for covariate factors. Very preterm infants are at a greater risk of developing socioemotional problems compared to term born infants, particularly in the domains of inattention-hyperactivity, anxiety, emotional disturbance, and social difficulties (55). Morphine exposure and painful NICU procedures in the absence of analgesia have been reported to be associated with increased parental reports of behavioral problems (16, 56). Our findings confirm a high risk of depression in survivors of neonatal intensive care; however, fentanyl does not appear to be a driver of this risk. Post-discharge environmental influences on the risk for developing later socioemotional problems in very preterm infants have been more extensively studied than the impact of cumulative fentanyl dose. Positive parental relations, such as greater sensitivity and non-hostility, and lower reports of parenteral stress have been associated with reduced internalizing behaviors in very preterm infants at 18 months corrected age (57). Conversely, controlling parenting styles, maternal depression, and reduced perception of family coherence have been associated with increased risk of emotional-behavioral problems (58, 59). While it is a unique finding that fentanyl was associated with decreased caregiver reported socioemotional problems, the impact of family dysfunction on socioemotional problems at 5 years of age is also notable.

Cumulative fentanyl dose was not independently associated with full scale intelligence quotients, language ability, or executive function skills at 5 years in our cohort which is consistent with previous findings (22, 24, 25). Despite the lack of adverse neurodevelopmental findings of our study, it must be acknowledged that this cohort was exposed to high cumulative doses of fentanyl throughout their neonatal course in comparison to other very preterm cohorts (24, 25). More recent literature regarding the pharmacokinetics of fentanyl in very preterm infants has emerged (60). Based on these pharmacokinetic models, the initial dosing strategy for continuous infusions in our cohort likely resulted in supratherapeutic fentanyl concentrations. Although our findings suggested that higher cumulative fentanyl dose was not independently associated with adverse long-term neurodevelopmental effects, these findings should not be interpreted as establishing a safe cumulative dose of fentanyl in very preterm infants. Further investigation of safe dosing requires prospective evaluation utilizing appropriate dosing based on robust pharmacokinetic studies.

Our study is limited by its observational nature and relatively small size. Additionally, we recognize that current pain management protocols in the NICU have evolved since the time in which patients were enrolled, which may limit the generalizability of our findings. While we were able to detect correlations, we cannot directly attribute cumulative fentanyl dose causatively to our findings. We acknowledge that pain scores were not collected as part of routine clinical care in our NICU at the time of fentanyl administration, so we are unable to assess the appropriateness of fentanyl administration in the setting of pain. Furthermore, cumulative fentanyl dose may have been a surrogate marker for the severity of illness and could be confounded by indication. Therefore, we do not feel the results of our study should encourage or discourage utilization of fentanyl in clinical practice and refer readers to previous topical reviews (61). Our patient population was also at risk for bias based on the ability of participants to follow-up, although our follow-up rates are high compared to many other similar longitudinal cohort studies.

## Conclusion

Cumulative fentanyl dose at term corrected age did not correlate with 5-year neurodevelopmental outcomes when adjusting for other variables. Cumulative fentanyl dose and family dysfunction were associated with caregiver reports of socioemotional problems on composite CBCL/1.5-5 Total Problem scores, which persisted after adjusting for covariate factors. Large, prospective studies and/or randomized controlled trials of analgesic and sedative exposure with long-term follow-up are warranted to better understand the safety of analgesia in very preterm infants.

## Data Availability Statement

The original contributions presented in the study are included in the article/Supplementary Material, further inquiries can be directed to the corresponding author.

## Ethics Statement

The studies involving human participants were reviewed and approved by Washington University Institutional Review Board Washington University School of Medicine in St. Louis. Written informed consent to participate in this study was provided by the participants' legal guardian/next of kin.

## Author Contributions

CS, TI, CR, and CM designed the study. RL and CM organized the database. KM performed the statistical analysis and wrote the first draft of the manuscript. All authors contributed to the article and approved the submitted version.

## Funding

Support by the National Institute of Health (R01 HD057098 to TI; R01 MH113570 to CR and CS; K01 MH122735 to RL; K02 NS089852 to CS; K12 HD055931-06; K23 MH105179 to CR), the Intellectual and Developmental Disabilities Research Center at Washington University (NIH/NICHD P50 HD103525), and the Doris Duke Charitable Foundation.

## Conflict of Interest

The authors declare that the research was conducted in the absence of any commercial or financial relationships that could be construed as a potential conflict of interest.

## Publisher's Note

All claims expressed in this article are solely those of the authors and do not necessarily represent those of their affiliated organizations, or those of the publisher, the editors and the reviewers. Any product that may be evaluated in this article, or claim that may be made by its manufacturer, is not guaranteed or endorsed by the publisher.

## Supplementary Material

Supplementary Figure 1 Log of cumulative Fentanyl Dose in Relation to CBCL/1-1.5 Affective Problems T-score. Center line represents fitted least-squares regression line. CBCL/1-1.5, Child Behavior Checklist/1.5-5.

Supplementary Table 1 All variables evaluated on bivariate analysis in association with MABC-2 Total t-score. CBH, cerebellar hemorrhage; CRIB, Clinical Risk Index for Babies; IVH, intraventricular hemorrhage; MRI, magnetic resonance image; PDA, patent ductus arteriosus; PVL, periventricular leukomalacia; TPN, total parenteral nutrition.

Supplementary Table 2 All CBCL/1.5-5 subscale T-scores in relation to log of cumulative fentanyl dose on bivariate linear regression. ADHD, Attention-deficit/hyperactivity disorder.

Supplementary Table 3 All variables evaluated on bivariate analysis in association with CBCL/1.5-5 Total Problems t-score. CBH, cerebellar hemorrhage; CRIB, Clinical Risk Index for Babies; IVH, intraventricular hemorrhage; MRI, magnetic resonance image; PDA, patent ductus arteriosus; PVL, periventricular leukomalacia; TPN, total parenteral nutrition.

## References

[1] Fitzgerald M. The development of nociceptive circuits. Nat Rev Neurosci. (2005) 6:507–20. 10.1038/nrn1701 15995722

[2] Anand KJ. Effects of perinatal pain and stress. Prog Brain Res. (2000) 122:117–29. 10.1016/S0079-6123(08)62134-2 10737054

[3] Fleiss B Gressens P. Neuroprotection of the preterm brain. Handb Clin Neurol. (2019) 162:315–28. 10.1016/B978-0-444-64029-1.00015-1 31324318

[4] Smith GC Gutovich J Smyser C Pineda R Newnham C Tjoeng TH. Neonatal intensive care unit stress is associated with brain development in preterm infants. Ann Neurol. (2011) 70:541–9. 10.1002/ana.22545 21976396 PMC4627473

[5] Ranger M Zwicker JG Chau CM Park MT Chakravarthy MM Poskitt K. Neonatal pain and infection relate to smaller cerebellum in very preterm children at school age. J Pediatr. (2015) 167:292–8.e1. 10.1016/j.jpeds.2015.04.055 25987534

[6] Vinall J Miller SP Bjornson BH Fitzpatrick KP Poskitt KJ Brant R. Invasive procedures in preterm children: brain and cognitive development at school age. Pediatrics. (2014) 133:412–21. 10.1542/peds.2013-1863 24534406 PMC3934331

[7] Aranda JV Carlo W Hummel P Thomas R Lehr VT Anand KJ. Analgesia and sedation during mechanical ventilation in neonates. Clin Ther. (2005) 27:877–99. 10.1016/j.clinthera.2005.06.019 16117990

[8] Taddio A Pulleyblank R Stephens D McNair C Shah V. Canadian neonatologist practices regarding opioid use in ventilated and spontaneously breathing infants undergoing medical procedures. Clin J Pain. (2010) 26:422–8. 10.1097/AJP.0b013e3181d36da7 20473050

[9] Hall RW Kronsberg SS Barton BA Kaiser JR Anand KJ NEOPAIN Trial Investigators Group. Morphine, hypotension, and adverse outcomes among preterm neonates: who's to blame? Secondary results from the NEOPAIN trial. Pediatrics. (2005) 115:1351–9. 10.1542/peds.2004-1398 15867047

[10] Anand KJ Barton BA McIntosh N Lagercrantz H Pelausa E Young TE. Analgesia and sedation in preterm neonates who require ventilatory support: results from the NOPAIN trial. Neonatal outcome and prolonged analgesia in neonates. Arch Pediatr Adolesc Med. (1999) 153:331–8. 10.1001/archpedi.153.4.331 10201714

[11] Anand KJ Hall RW Desai N Shephard B Bergqvist LL Young TE. Effects of morphine analgesia in ventilated preterm neonates: primary outcomes from the NEOPAIN randomised trial. Lancet. (2004) 363:1673–82. 10.1016/S0140-6736(04)16251-X 15158628

[12] Simons SH van Dijk M van Lingen RA Roofthooft D Duivenvoorden HJ Jongeneel N. Routine morphine infusion in preterm newborns who received ventilatory support: a randomized controlled trial. JAMA. (2003) 290:2419–27. 10.1001/jama.290.18.2419 14612478

[13] Steinhorn R McPherson C Anderson PJ Neil J Doyle LW Inder T. Neonatal morphine exposure in very preterm infants-cerebral development and outcomes. J Pediatr. (2015) 166:1200–7.e4. 10.1016/j.jpeds.2015.02.012 25919729 PMC4928575

[14] de Graaf J van Lingen RA Valkenburg AJ Weisglas-Kuperus N Jebbink LG Wijnberg-Williams B. Does neonatal morphine use affect neuropsychological outcomes at 8 to 9 years of age? Pain. (2013) 154:449–58. 10.1016/j.pain.2012.12.006 23352760

[15] Ferguson SA Ward WL Paule MG Hall RW Anand KJ. A pilot study of preemptive morphine analgesia in preterm neonates: effects on head circumference, social behavior, and response latencies in early childhood. Neurotoxicol Teratol. (2012) 34:47–55. 10.1016/j.ntt.2011.10.008 22094261

[16] Zwicker JG Miller SP Grunau RE Chau V Brant R Studholme C. Smaller cerebellar growth and poorer neurodevelopmental outcomes in very preterm infants exposed to neonatal morphine. J Pediatr. (2016) 172:81–7.e2. 10.1016/j.jpeds.2015.12.024 26763312 PMC5462546

[17] Saarenmaa E Huttunen P Leppäluoto J Meretoja O Fellman V. Advantages of fentanyl over morphine in analgesia for ventilated newborn infants after birth: a randomized trial. J Pediatr. (1999) 134:144–50. 10.1016/S0022-3476(99)70407-5 9931520

[18] Walter-Nicolet E Annequin D Biran V Mitanchez D Tourniaire B. Pain management in newborns: from prevention to treatment. Paediatr Drugs. (2010) 12:353–65. 10.2165/11318900-000000000-00000 21028915

[19] Ancora G Lago P Garetti E Pirelli A Merazzi D Mastrocola M. Efficacy and safety of continuous infusion of fentanyl for pain control in preterm newborns on mechanical ventilation. J Pediatr. (2013) 163:645–51.e1. 10.1016/j.jpeds.2013.02.039 23582138

[20] Lago P Benini F Agosto C Zacchello F. Randomised controlled trial of low dose fentanyl infusion in preterm infants with hyaline membrane disease. Arch Dis Child Fetal Neonatal Ed. (1998) 79:F194–7. 10.1136/fn.79.3.F194 10194990 PMC1720853

[21] Orsini AJ Leef KH Costarino A Dettorre MD Stefano JL. Routine use of fentanyl infusions for pain and stress reduction in infants with respiratory distress syndrome. J Pediatr. (1996) 129:140–5. 10.1016/S0022-3476(96)70201-9 8757574

[22] McPherson C Haslam M Pineda R Rogers C Neil JJ Inder TE. Brain injury and development in preterm infants exposed to fentanyl. Ann Pharmacother. (2015) 49:1291–7. 10.1177/1060028015606732 26369570 PMC4644677

[23] Durrmeyer X Vutskits L Anand KJ Rimensberger PC. Use of analgesic and sedative drugs in the NICU: integrating clinical trials and laboratory data. Pediatr Res. (2010) 67:117–27. 10.1203/PDR.0b013e3181c8eef3 20091937

[24] Lammers EM Johnson PN Ernst KD Hagemann TM Lawrence SM Williams PK. Association of fentanyl with neurodevelopmental outcomes in very-low-birth-weight infants. Ann Pharmacother. (2014) 48:335–42. 10.1177/1060028013514026 24311724

[25] Ancora G Lago P Garetti E Pirelli A Merazzi D Pierantoni L. Follow-up at the corrected age of 24 months of preterm newborns receiving continuous infusion of fentanyl for pain control during mechanical ventilation. Pain. (2017) 158:840–5. 10.1097/j.pain.0000000000000839 28240994

[26] Zmyj N Witt S Weitkämper A Neumann H Lücke T. Social cognition in children born preterm: a perspective on future research directions. Front Psychol. (2017) 8:455. 10.3389/fpsyg.2017.00455 28611695 PMC5447081

[27] de Courcy-Wheeler RH Wolfe CD Fitzgerald A Spencer M Goodman JD Gamsu HR. Use of the CRIB (clinical risk index for babies) score in prediction of neonatal mortality and morbidity. Arch Dis Child Fetal Neonatal Ed. (1995) 73:F32–6. 10.1136/fn.73.1.F32 7552593 PMC2528363

[28] Lean RE Paul RA Smyser TA Smyser CD Rogers CE. Social adversity and cognitive, language, and motor development of very preterm children from 2 to 5 years of age. J Pediatr. (2018) 203:177–84.e1. 10.1016/j.jpeds.2018.07.110 30244986 PMC6252144

[29] Epstein NB Baldwin LM Bishop D. The mcmaster family assessment device. J Marital Family Ther. (1983) 9:171–80. 10.1111/j.1752-0606.1983.tb01497.x

[30] Wechsler D. WPPSI-III: Administration and Scoring Manual. San Antonio, TX: The Psychological Corporation (2004).

[31] Semel E Wiig EH Secord WA. Clinical Evaluation of Language Fundamentalls Preschool. 2nd Ed. San Antonio, TX: Harcourt Assessment PsyCorp (2004).

[32] Henderson SE Sugden DA Barnett AL. Movement Assessment Battery for Children-2: Movement ABC-2: Examiner's Manual. São Paulo: Pearson (2007).

[33] Gordon B. Test Review: Wechsler D. The Wechsler Preschool and Primary Scale of Intelligence, Third Edition (WPPSI-III). San Antonio, TX: The Psychological Corporation - ProQuest. (2004). p. 205–20.

[34] Davis NM Ford GW Anderson PJ Doyle LW Victorian Infant Collaborative Study Group. Developmental coordination disorder at 8 years of age in a regional cohort of extremely-low-birthweight or very preterm infants. Dev Med Child Neurol. (2007) 49:325–30. 10.1111/j.1469-8749.2007.00325.x 17489804

[35] Spencer EJ Spencer TD Goldstein H Schneider N. Identifying early literacy learning needs. In: Shanahan T Lonigan CJ editors. Early Childhood Literacy: The National Early Literacy Panel and Beyond. 1st ed. Baltimore, MD: Brookes Publishing (2013). p. 45–70.

[36] Nieto M Ros L Medina G Ricarte JJ Latorre JM. Assessing executive functions in preschoolers using shape school task. Front Psychol. (2016) 7:1489. 10.3389/fpsyg.2016.01489 27729896 PMC5037173

[37] Espy KA Bull R Martin J Stroup W. Measuring the development of executive control with the shape school. Psychol Assess. (2006) 18:373–81. 10.1037/1040-3590.18.4.373 17154758

[38] Lean RE Gerstein ED Smyser TA Smyser CD Rogers CE. Socioeconomic disadvantage and parental mood/affective problems links negative parenting and executive dysfunction in children born very preterm. Dev Psychopathol. (2021) 1–16. 10.1017/S0954579421000961. [Epub ahead of print]. 34725016 PMC9058043

[39] Pritchard VE Woodward LJ. Preschool executive control on the shape school task: measurement considerations and utility. Psychol Assess. (2011) 23:31–43. 10.1037/a0021095 21381841

[40] Achenbach TM Roescorla LA. Manual for the Aseba School-Age Forms & Profiles. University of Vermont, Research Center for Children, Youth, & Families, Burlington, Canada (2001).

[41] Constantino JN Gruber CP. Social Responsiveness Scale. 2nd ed. (Srs-2). Torrance, CA: Western Psychological Services (2012).

[42] Bruni TP. Test review: social responsiveness scale–second edition (SRS-2). J Psychoeduc Assess. (2014) 32:365–9. 10.1177/073428291351752533284813

[43] Warnick EM Bracken MB Kasl S. Screening efficiency of the child behavior checklist and strengths and difficulties questionnaire: a systematic review. Child Adol Mental Health. (2008) 13:140–7. 10.1111/j.1475-3588.2007.00461.x 32847173

[44] Volpe JJ Inder TE Darras BT de Vries LS du Plessis AJ Neil J. Volpe's Neurology of the Newborn. 6th Ed. Philadelphia, PA: Elsevier (2018).

[45] Kidokoro H Neil JJ Inder TE. New MR imaging assessment tool to define brain abnormalities in very preterm infants at term. AJNR Am J Neuroradiol. (2013) 34:2208–14. 10.3174/ajnr.A3521 23620070 PMC4163698

[46] Hammer RP Jr Ricalde AA Seatriz JV. Effects of opiates on brain development. Neurotoxicology. (1989) 10:475–83. 2696899

[47] Hauser KF Gurwell JA Turbek CS. Morphine inhibits purkinje cell survival and dendritic differentiation in organotypic cultures of the mouse cerebellum. Exp Neurol. (1994) 130:95–105. 10.1006/exnr.1994.1188 7821399 PMC4306355

[48] Koziol LF Budding D Andreasen N D'Arrigo S Bulgheroni S Imamizu H. Consensus paper: the cerebellum's role in movement and cognition. Cerebellum. (2014) 13:151–77. 10.1007/s12311-013-0511-x 23996631 PMC4089997

[49] Limperopoulos C Bassan H Gauvreau K Robertson RL Jr Sullivan NR Benson CB. Does cerebellar injury in premature infants contribute to the high prevalence of long-term cognitive, learning, and behavioral disability in survivors? Pediatrics. (2007) 120:584–93. 10.1542/peds.2007-1041 17766532

[50] Limperopoulos C Soul JS Haidar H Huppi PS Bassan H Warfield SK. Impaired trophic interactions between the cerebellum and the cerebrum among preterm infants. Pediatrics. (2005) 116:844–50. 10.1542/peds.2004-2282 16199692

[51] Volpe JJ. Cerebellum of the premature infant: rapidly developing, vulnerable, clinically important. J Child Neurol. (2009) 24:1085–104. 10.1177/0883073809338067 19745085 PMC2799249

[52] de Vries AM de Groot L. Transient dystonias revisited: a comparative study of preterm and term children at 2 1/2 years of age. Dev Med Child Neurol. (2002) 44:415–21. 10.1017/S0012162201002298 12088310

[53] de Kleine MJ den Ouden AL Kollée LA Nijhuis-van der Sanden MW Sondaar M van Kessel-Feddema BJ. Development and evaluation of a follow up assessment of preterm infants at 5 years of age. Arch Dis Child. (2003) 88:870–5. 10.1136/adc.88.10.870 14500304 PMC1719302

[54] Erikson C Allert C Carlberg EB Katz-Salamon M. Stability of longitudinal motor development in very low birthweight infants from 5 months to 5.5 years. Acta Paediatr. (2003) 92:197–203. 10.1111/j.1651-2227.2003.tb00526.x 12710646

[55] Delobel-Ayoub M Arnaud C White-Koning M Casper C Pierrat V Garel M. Behavioral problems and cognitive performance at 5 years of age after very preterm birth: the EPIPAGE study. Pediatrics. (2009) 123:1485–92. 10.1542/peds.2008-1216 19482758

[56] Ranger M Synnes AR Vinall J Grunau RE. Internalizing behaviours in school-age children born very preterm are predicted by neonatal pain and morphine exposure. Eur J Pain. (2014) 18:844–52. 10.1002/j.1532-2149.2013.00431.x 24318537 PMC4016156

[57] Vinall J Miller SP Synnes AR Grunau RE. Parent behaviors moderate the relationship between neonatal pain and internalizing behaviors at 18 months corrected age in children born very prematurely. Pain. (2013) 154:1831–9. 10.1016/j.pain.2013.05.050 23748079 PMC3791108

[58] Forcada-Guex M Pierrehumbert B Borghini A Moessinger A Muller-Nix C. Early dyadic patterns of mother-infant interactions and outcomes of prematurity at 18 months. Pediatrics. (2006) 118:e107–14. 10.1542/peds.2005-1145 16818525

[59] Huhtala M Korja R Lehtonen L Haataja L Lapinleimu H Rautava P. Associations between parental psychological well-being and socio-emotional development in 5-year-old preterm children. Early Hum Dev. (2014) 90:119–24. 10.1016/j.earlhumdev.2013.12.009 24418104

[60] Völler S Flint RB Andriessen P Allegaert K Zimmermann LJI Liem KD. Rapidly maturing fentanyl clearance in preterm neonates. Arch Dis Child Fetal Neonatal Ed. (2019) 104:F598–603. 10.1136/archdischild-2018-315920 31498775

[61] McPherson C Ortinau CM Vesoulis Z. Practical approaches to sedation and analgesia in the newborn. J Perinatol. (2021) 41:383–95. 10.1038/s41372-020-00878-7 33250515 PMC7700106

