# Supplementary figures and images for: Fentanyl Exposure in Preterm Infants: Five-Year Neurodevelopmental and Socioemotional Assessment
Source: Front Pain Res (Lausanne). 2022 Mar 1;3:836705. doi: 10.3389/fpain.2022.836705 (PMC9429367; doi:10.3389/fpain.2022.836705)

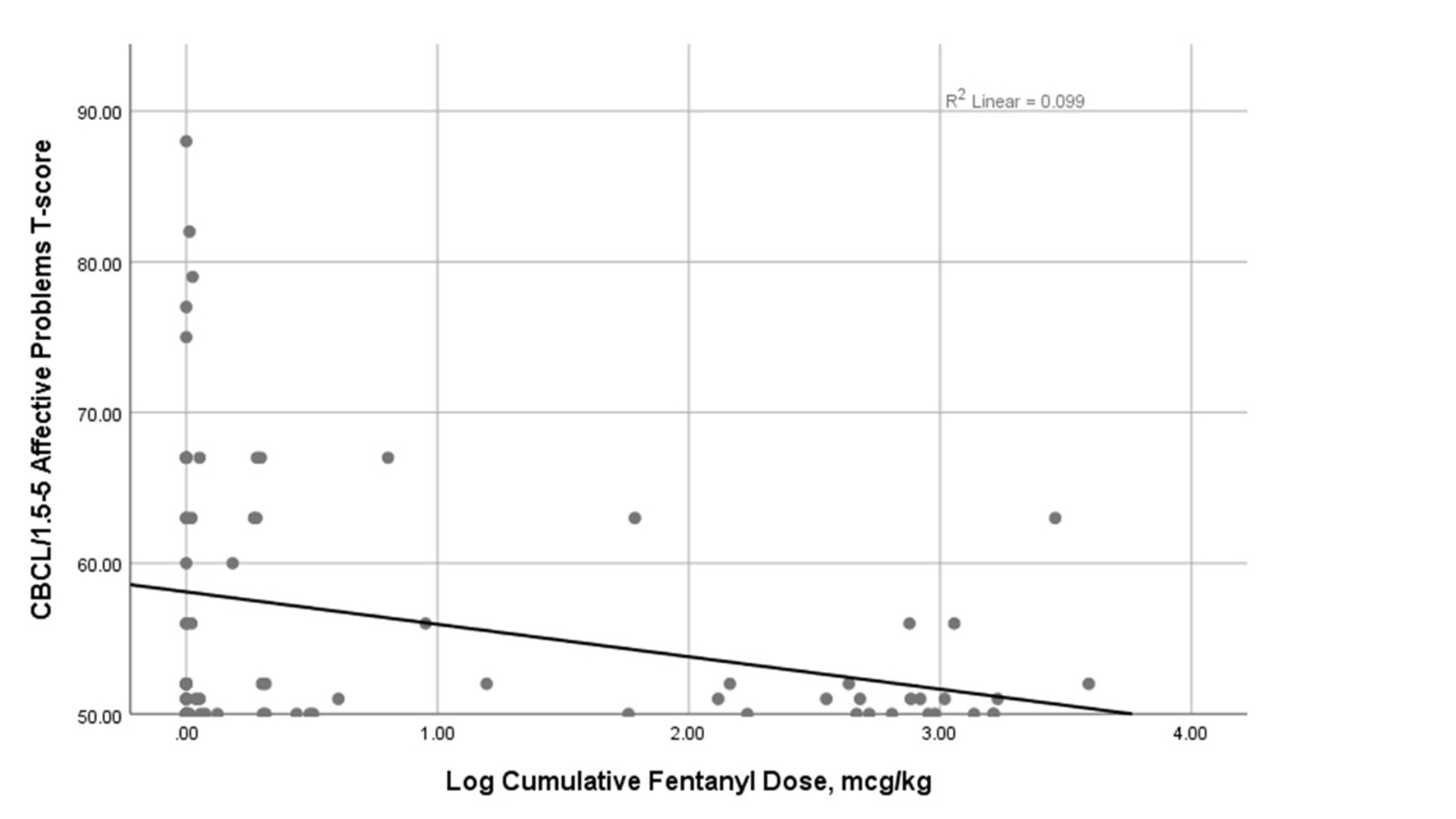

Supplement: Supplementary Figure 1 — Log of cumulative Fentanyl Dose in Relation to CBCL/1-1.5 Affective Problems T-score. Center line represents fitted least-squares regression line. CBCL/1-1.5, Child Behavior Checklist/1.5-5. [file Image_1.jpg]
