# Supplementary material for: Fentanyl Exposure in Preterm Infants: Five-Year Neurodevelopmental and Socioemotional Assessment
Source: Front Pain Res (Lausanne). 2022 Mar 1;3:836705. doi: 10.3389/fpain.2022.836705 (PMC9429367; doi:10.3389/fpain.2022.836705)
Supplement: Supplementary Table 1 — All variables evaluated on bivariate analysis in association with MABC-2 Total t-score. CBH, cerebellar hemorrhage; CRIB, Clinical Risk Index for Babies; IVH, intraventricular hemorrhage; MRI, magnetic resonance image; PDA, patent ductus arteriosus; PVL, periventricular leukomalacia; TPN, total parenteral nutrition. [file Table_1.docx]

Supplemental Table 1. All variables evaluated on bivariate analysis in association with MABC-2 Total t-score

| Variable | B | SE | R2 | P value |
| --- | --- | --- | --- | --- |
| Maternal age | 0.241 | 0.165 | 0.028 | 0.148 |
| Antenatal steroids | 1.071 | 4.239 | 0.001 | 0.801 |
| Gestational age | 1.54 | 0.682 | 0.065 | **0.027** |
| Birthweight | 0.016 | 0.005 | 0.142 | **0.001** |
| Sex | 0.896 | 2.465 | 0.002 | 0.717 |
| 5-minute APGAR score | 0.787 | 0.596 | 0.023 | 0.191 |
| CRIB score | -0.926 | 0.348 | 0.089 | **0.009** |
| Log ventilation days | -2.955 | 0.75 | 0.175 | **<0.0001** |
| Log TPN days | -2.151 | 1.75 | 0.02 | 0.223 |
| PDA requiring treatment | -2.884 | 2.455 | 0.019 | 0.244 |
| Necrotizing enterocolitis | -0.978 | 4.546 | 0.001 | 0.83 |
| Retinopathy of prematurity | -7.983 | 5.055 | 0.06 | 0.122 |
| Chronic lung disease | 2.128 | 3.481 | 0.009 | 0.545 |
| IVH on cranial ultrasound | -1.48 | 1.161 | 0.023 | 0.206 |
| PVL on cranial ultrasound | -3.248 | 2.382 | 0.026 | 0.177 |
| CBH on MRI | -4.579 | 3.070 | 0.031 | 0.14 |
| Cerebellum diameter | 0.416 | 0.422 | 0.049 | 0.337 |
| Log anesthesia hours | -1.13 | 0.625 | 0.046 | **0.075** |
| Log inotrope hours | -1.376 | 0.792 | 0.072 | **0.09** |
| Log morphine dose | -9.553 | 5.819 | 0.036 | 0.105 |
| Log midazolam dose | -9.274 | 4.856 | 0.048 | **0.06** |
| Log dexamethasone dose | -14.588 | 9.986 | 0.028 | 0.148 |
| Log hydrocortisone dose | -2.009 | 0.789 | 0.083 | **0.013** |
| Social risk composite | -0.64 | 0.928 | 0.006 | 0.493 |
| Income to needs ratio | 0.355 | 0.594 | 0.005 | 0.553 |
| McMaster Family Assessment Device | 0.088 | 3.235 | <0.0001 | 0.978 |

CBH = cerebellar hemorrhage; CRIB = Clinical Risk Index for Babies; IVH = intraventricular hemorrhage; MRI = magnetic resonance image; PDA = patent ductus arteriosus; PVL = periventricular leukomalacia; TPN = total parenteral nutrition
