# Supplementary material for: Fentanyl Exposure in Preterm Infants: Five-Year Neurodevelopmental and Socioemotional Assessment
Source: Front Pain Res (Lausanne). 2022 Mar 1;3:836705. doi: 10.3389/fpain.2022.836705 (PMC9429367; doi:10.3389/fpain.2022.836705)
Supplement: Supplementary Table 2 — All CBCL/1.5-5 subscale T-scores in relation to log of cumulative fentanyl dose on bivariate linear regression. ADHD, Attention-deficit/hyperactivity disorder. [file Table_2.DOCX]

Supplemental Table 2. All CBCL/1.5-5 subscale T-scores in relation to log of cumulative fentanyl dose on bivariate linear regression

| Variable | B | SE | R2 | P value |
| --- | --- | --- | --- | --- |
| Stress Problems t-score | -1.230 | 0.692 | 0.039 | 0.08 |
| Affective Problems t-score | -2.152 | 0.745 | 0.099 | **0.005** |
| Anxiety Problems t-score | -1.169 | 0.785 | 0.028 | 0.141 |
| Pervasive Developmental Problems t-score | -1.335 | 0.906 | 0.028 | 0.145 |
| ADHD Problems t-score | -0.693 | 0.791 | 0.01 | 0.384 |
| Oppositional Defiant Problems t-score | -1.232 | 0.626 | 0.048 | 0.053 |

ADHD = Attention-deficit/hyperactivity disorder
