# Supplementary material for: Fentanyl Exposure in Preterm Infants: Five-Year Neurodevelopmental and Socioemotional Assessment
Source: Front Pain Res (Lausanne). 2022 Mar 1;3:836705. doi: 10.3389/fpain.2022.836705 (PMC9429367; doi:10.3389/fpain.2022.836705)
Supplement: Supplementary Table 3 — All variables evaluated on bivariate analysis in association with CBCL/1.5-5 Total Problems t-score. CBH, cerebellar hemorrhage; CRIB, Clinical Risk Index for Babies; IVH, intraventricular hemorrhage; MRI, magnetic resonance image; PDA, patent ductus arteriosus; PVL, periventricular leukomalacia; TPN, total parenteral nutrition. [file Table_3.docx]

Supplemental Table 3. All variables evaluated on bivariate analysis in association with CBCL/1.5-5 Total Problems t-score

| Variable | B | SE | R2 | P value |
| --- | --- | --- | --- | --- |
| Maternal age | -0.576 | 0.224 | 0.079 | **0.012** |
| Antenatal steroids | -6.529 | 5.224 | 0.02 | 0.215 |
| Gestational age | 1.684 | 0.968 | 0.038 | **0.086** |
| Birthweight | 0.01 | 0.007 | 0.025 | 0.163 |
| Sex | 0.197 | 3.509 | <0.0001 | 0.955 |
| 5-minute APGAR score | 0.835 | 0.858 | 0.012 | 0.339 |
| CRIB score | -0.387 | 0.516 | 0.007 | 0.456 |
| Log ventilation days | -0.344 | 1.187 | 0.001 | 0.773 |
| Log TPN days | -4.945 | 2.4 | 0.052 | **0.043** |
| PDA requiring treatment | -6.007 | 3.456 | 0.038 | **0.086** |
| Necrotizing enterocolitis | -8.324 | 7.143 | 0.017 | 0.247 |
| Retinopathy of prematurity | 3.974 | 10.347 | 0.004 | 0.703 |
| Chronic lung disease | -6.398 | 5.344 | 0.035 | 0.238 |
| IVH on cranial ultrasound | 0.028 | 1.401 | <0.0001 | 0.984 |
| PVL on cranial ultrasound | 2.293 | 2.505 | 0.011 | 0.363 |
| CBH on MRI | -0.854 | 4.436 | 0.001 | 0.848 |
| Cerebellum diameter | -0.866 | 0.816 | 0.056 | 0.301 |
| Log anesthesia hours | -1.206 | 0.849 | 0.028 | 0.16 |
| Log inotrope hours | -2.783 | 1.211 | 0.117 | **0.027** |
| Log morphine dose | 4.759 | 6.358 | 0.007 | 0.456 |
| Log midazolam dose | 1.572 | 5.031 | 0.001 | 0.755 |
| Log dexamethasone dose | 2.849 | 14.767 | <0.0001 | 0.848 |
| Log hydrocortisone dose | -0.213 | 1.205 | <0.0001 | 0.86 |
| Social risk composite | 1.101 | 1.313 | 0.009 | 0.404 |
| Income to needs ratio | -0.151 | 0.854 | <0.0001 | 0.86 |
| McMaster Family Assessment Device | 14.977 | 4.089 | 0.157 | **<0.0001** |

CBH = cerebellar hemorrhage; CRIB = Clinical Risk Index for Babies; IVH = intraventricular hemorrhage; MRI = magnetic resonance image; PDA = patent ductus arteriosus; PVL = periventricular leukomalacia; TPN = total parenteral nutrition
